# iNOS‐inhibitor driven neuroprotection in a porcine retina organ culture model

**DOI:** 10.1111/jcmm.15091

**Published:** 2020-03-04

**Authors:** José Hurst, Ana Maria Mueller‐Buehl, Lisa Hofmann, Sandra Kuehn, Fenja Herms, Sven Schnichels, Stephanie Christine Joachim

**Affiliations:** ^1^ Centre for Ophthalmology Tübingen University Eye Hospital Tübingen Germany; ^2^ Experimental Eye Research Institute University Eye Hospital Ruhr‐University Bochum Bochum Germany

**Keywords:** hypoxia, iNOS‐inhibitor 1400W, organ culture, retina, retinal ganglion cells

## Abstract

Nitrite oxide plays an important role in the pathogenesis of various retinal diseases, especially when hypoxic processes are involved. This degeneration can be simulated by incubating porcine retinal explants with CoCl_2_. Here, the therapeutic potential of iNOS‐inhibitor 1400W was evaluated. Degeneration through CoCl_2_ and treatment with the 1400W were applied simultaneously to porcine retinae explants. Three groups were compared: control, CoCl_2_, and CoCl_2_ + iNOS‐inhibitor (1400W). At days 4 and 8, retinal ganglion cells (RGCs), bipolar, and amacrine cells were analysed. Furthermore, the influence on the glia cells and different stress markers were evaluated. Treatment with CoCl_2_ resulted in a significant loss of RGCs already after 4 days, which was counteracted by the iNOS‐inhibitor. Expression of HIF‐1α and its downstream targets confirmed the effective treatment with 1400W. After 8 days, the CoCl_2_ group displayed a significant loss in amacrine cells and also a drastic reduction in bipolar cells was observed, which was prevented by 1400W. The decrease in microglia could not be prevented by the inhibitor. CoCl_2_ induces strong degeneration in porcine retinae by mimicking hypoxia, damaging certain retinal cell types. Treatment with the iNOS‐inhibitor counteracted these effects to some extent, by preventing loss of retinal ganglion and bipolar cells. Hence, this inhibitor seems to be a very promising treatment for retinal diseases.

## 1 INTRODUCTION

Since 1995, 11 million animals per year were used for research purposes in the EU, an average of 2.5 million in Germany alone, with an upward trend.^1^ Ophthalmological research also frequently uses laboratory animals. An important field of research is the understanding of retinal degeneration, as its causes are not yet fully understood preventing successful treatment.

In the last years, we developed retinal organ culture models, to test new therapies without euthanasia of laboratory animals.^2^, ^3^, ^4^, ^5^ These models are based on porcine retinae, which can be obtained as a waste product from the food industry. The morphology and physiology of the porcine retina is very similar to that of the human retina.^6^

The retina is known to be extremely sensitive to fluctuations in oxygen levels and hypoxia is known to cause development of retinopathy and retinal degenerative diseases. Common to all retinal degenerative diseases is the deterioration of the retina caused by the progressive degeneration and death of the different retinal cells. For example, there is evidence for a causal link between oxidative stress and age‐related macular degeneration (AMD).^7^, ^8^, ^9^, ^10^ Several publications indicate that oxidative stress and ischaemia, an early event, which occurs under the high ocular pressure present in many forms of glaucoma, induces retinal ganglion cells (RGC) damage.^11^, ^12^, ^13^, ^14^, ^15^ Vascular occlusions of the retina, including arterial and venous obstructions, are among the most frequent causes of vision loss.^16^ During ischaemia of the inner retina, a large number of different cell types are affected, out of those the RGC represent the most sensitive population, usually die first.^17^ As with all neurons, regeneration is not possible. Even worse, the preservation of damaged cells is very difficult due to the environment and the signals generated by the surrounding tissue (eg glia cells). Hence, there is an urgent need to develop new and more effective therapeutic strategies to combat these devastating diseases. In order to be able to find new treatment approaches for these diseases, models with which pathophysiology can be simulated are necessary. Hence, an ex vivo model for retinal hypoxia in pig retina was developed.^11^ The treatment of retinal explants with cobalt chloride (CoCl_2_) induces degeneration in the target tissue corresponding to the clinical picture of ischaemic retinopathies. In the here presented study, the protective effect of the inducible nitric oxide synthase (iNOS) inhibitor 1400W were investigated. Cytokine‐inducible nitric oxide synthase is an immune regulator in the retina and mainly found in Müller cells and in retinal pigment epithelium.^18^ iNOS is induced under pathological conditions by endotoxins, inflammation, and cytokines and causes pathophysiological reactions leading to optic nerve and retinal degeneration.^8^ It is involved in phagocytosis during infectious and ischaemic processes. Once induced iNOS produces large amounts of nitric oxide (NO).^19^ Nitric oxide is an essential signalling molecule, which plays a role in neurotransmission, host cell defence and vasodilation.^20^, ^21^ There are three isoforms of the nitric oxide synthase (NOS), the enzyme that produces NO, neuronal, immunologic and endothelial isoform. The first two are present in the retina.^22^ The immunologic isoform is not constitutively expressed and requires induction usually by immunologic activation; calcium is not necessary for its activation as it is for the other two forms.^23^ Pathophysiological increase of NO through iNOS has major effects in all tissues, but especially in neuronal tissue, like the retina. NO mediates many of the destructive effects of interleukin (IL)‐1 in inflamed tissues. NO has been reported to activate matrix metalloproteinases,^24^ inhibit collagen synthesis and induce retinal apoptosis.^22^, ^25^ The resulting molecules nitrogen dioxide (NO_2_), nitrite, peroxynitrite and free radicals are responsible for a retinal degeneration that occurs in glaucoma, ischaemic retinopathies, and AMD.^19^

The inhibition of iNOS has been researched for years in cancer therapy and has also made its way into ophthalmology.^25^, ^26^, ^27^, ^28^ Here, we present a study investigating the effect of the iNOS‐inhibitor 1400W on retinal cells in the CoCl_2_ degeneration model 1400W. This iNOS‐inhibitor had a neuroprotective effect on neuronal cells in CoCl_2_ induced hypoxic degeneration model.

## 2 MATERIALS AND METHODS

### 2.1 CoCl_2_ induced degeneration model and treatment scheme

Retina explant preparation was performed as described previously.^2^ Briefly, porcine eyes were obtained from the local abattoir. Retina explants were cultured on inserts in 1 mL medium (Neurobasal‐A‐medium (Life Technologies) supplemented with 0.8 mmol/L l‐glutamine (Life Technologies), 2% B27 (Life Technologies), 1% N2 (Life Technologies) and 2% penicillin/streptomycin (Sigma‐Aldrich) in a six‐well plate (Millipore)).

**Figure 1:**
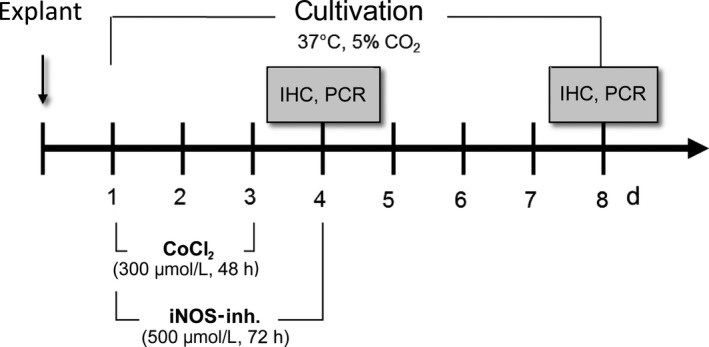
Procedure of the 1400W treatment in the CoCl_2_ degeneration model. CoCl_2_, cobalt chloride; IHC, immunohistochemistry; iNOS‐inh., iNOS‐inhibitor 1400W

For this study, a cultivation time of 4 and 8 days was chosen. Retina explants were exposed to CoCl_2_ from day 1 to day 3, for 48 hours (300 mmol/L; Figure 1). At the beginning of degeneration, treatment with 500 µmol/L iNOS‐inhibitor 1400W (Merck Millipore) was started simultaneously and lasted 72 hours until day 4. In preliminary studies, shorter time periods were also investigated, but they did not achieve the desired effects. Control retinas were cultivated continuously at 37°C without any treatment. The medium was exchanged completely on days 0, 1, 2 and 3 In addition, 50% medium were exchanged at day 6. On days 4 and 8, samples were frozen for subsequent the analyses immunohistochemistry and quantitative real‐time PCR (qRT‐PCR).

### 2.2 Histology

Fixation of retinal explants was performed for 15 minutes using 4% paraformaldehyde. Each explant was cryo‐protected with 15% sucrose for 15 minutes, 30% sucrose for 30 minutes, and then frozen in liquid nitrogen. Slides of 10 µm thick slices were cut using a cryostat.

### 2.3 Immunohistology

Retinal cross‐sections were pre‐incubated with blocking buffer containing 0.1%‐0.2% PBS/TritonX‐100 mixture (Merck Millipore) and 10%‐20% normal donkey serum (Dianova) for one hour. Primary antibodies (Table 1) were diluted in the blocking buffer and slices were incubated over night at room temperature. At the next day, retinal cross‐sections were incubated with fluorescence‐labelled secondary antibodies diluted in the same blocking buffer (Table 1). 4′,6′‐Diamidin‐2‐Phenylindol (DAPI, Dianova) was used to visualize cell nuclei.

**Table 1:** Primary antibodies used for histological analyses

Marker	Company	Dilution	Secondary antibody
RBPMS (rabbit)	Millipore	1:200	dαRb A555, 1:500, Invitrogen
HIF‐1α (mouse)	BD Bioscience	1:100	dαMs A488, 1:500, Invitrogen
Iba1 (rabbit)	WAKO	1:400	dαRb A555, 1:500, Invitrogen
Fcy‐R (CD16/32) (rat)	BD Bioscience	1:100	dαRat A555, 1:500, Thermo Fischer
Calretinin (goat)	Millipore	1:2000	dαgt A488, 1:500, Dianova
PKCα (mouse)	Santa Cruz	1:300	dαMs A488, 1:500, Invitrogen

#### 2.3.1 Immunohistological examination

Six retinal slices per explant were used for the evaluation. In the end, 24 masked images were counted for each staining. Cells were counted as positive, when the specific marker (RNA‐binding protein with multiple splicing [RBPMS], calretinin and Protein kinase C alpha [PKCα]) was co‐localized with DAPI. The total amount of microglia population was evaluated by counting all Ionized calcium‐binding adapter molecule 1 (Iba1^+^) and DAPI^+^ cells. Active microglia were counted when Fcy‐Rezeptor (Fcy‐R^+^) signals were additionally seen. All cell numbers are given in cells/mm.

### 2.4 Quantitative real‐time PCR

The expression of cell specific markers, like parvalbumin (*PVALB), CD11b and CC‐chemokine ligand 2 [CCL2]* were analysed. Markers of oxidative stress, including *iNOS,* hypoxia‐inducible factor (*HIF)‐1α and heat shock protein 70 (HSP70)*, were also investigated in the retinal organ model. mRNA was isolated from retinal explants and reverse transcribed using the MultiMACS mRNA and cDNA Synthesis Kit on the MultiMACS™ M96 Separator (Miltenyi Biotec) according to the manufacturer's protocol. After cDNA synthesis, qRT‐PCR was performed with 40 cycles using the Universal SYBR Green Supermix on a thermocycler (Biorad). About, 1 ng/µL of cDNA was used in a reaction volume of 20 µL according to the manufacturer's instructions. Final primer concentration was 100 nmol/L. The cDNA expression levels of the investigated genes were normalized to the cDNA level of the housekeeping genes RLP4 and β‐actin. The primers (Table 2) were designed using the Primer3 software (GenBank: KM035791.1, http://www.bioinformatics.nl/cgi-bin/primer3plus/primer3plus.cgi/).

**Table 2:** qRT‐PCR primer pairs in 5′‐3′ directions. The listed primer pairs were used in qRT‐PCR experiments, while *β‐actin* and *RLP4* were used as housekeeping genes

Gene	Oligonucleotide sequence
*HIF‐1α for*	TTACAGCAGCCAGATGATCG
*HIF‐1α rev*	TGGTCAGCTGTGGTAATCCA
*CD11b for*	AGAAGGAGACACCCAGAGCA
*CD11b rev*	GTAGGACAATGGGCGTCACT
*PVALB for*	CAACGCTGAGGACATCAAGA
*PVALB rev*	TGACAGGTCTCTGGCATCTG
*β‐Actin for*	CTCTTCCAGCCTTCCTTC
*β‐Actin rev*	GGGCAGTGATCTCTTTCT
*RPL4 for*	CAAGAGTAACTACAACCTTC
*RPL4 rev*	GAACTCTACGATGAATCTTC
*HSP70 for*	ATGTCCGCTGCAAGAGAAGT
*HSP70 rev*	GGCGTCAAACACGGTATTCT
*iNOS for*	TGTTCAGCTGTGCCTTCAAC
*iNOS2 rev*	CAGAACTGGGGGTACATGCT
*CCL2 for*	TCTCCAGTCACTGCTA
*CCL2 rev*	TCCAGGTGGCGGAGTC

Abbreviations: for, forward; rev, reverse.

### 2.5 Statistical analysis

In regard to immunohistological data, ANOVA followed by Tukey's post‐hoc test was applied to analyse differences between groups (Statistica, V 12). In accordance, qRT‐PCR data were analysed using ANOVA followed by Tukey's post‐hoc test to analyse differences between groups (GraphPad Prism 8). For all statistical tests, significance with respect to the control group is indicated using the following symbols and significance levels: *P < .05; **P < .01; ***P < .001.

## 3 RESULTS

### 3.1 Effect of 1400W on oxidative stress in retinal organ cultures

First, it was examined whether the iNOS‐inhibitor in the retinal organ cultures develops its effect via testing the protein and mRNA expression of the hypoxia marker HIF‐1‐α. As previously described, induction with CoCl_2_ leads to an increase in oxidative stress and activates transcription of HIF‐1α.^3^, ^4^ This is a specific oxygen‐sensitive subunit that regulates the activity of the transcription factor HIF‐1, which increases after ischaemia and can either promote or prevent neuronal survival.

**Figure 2:**
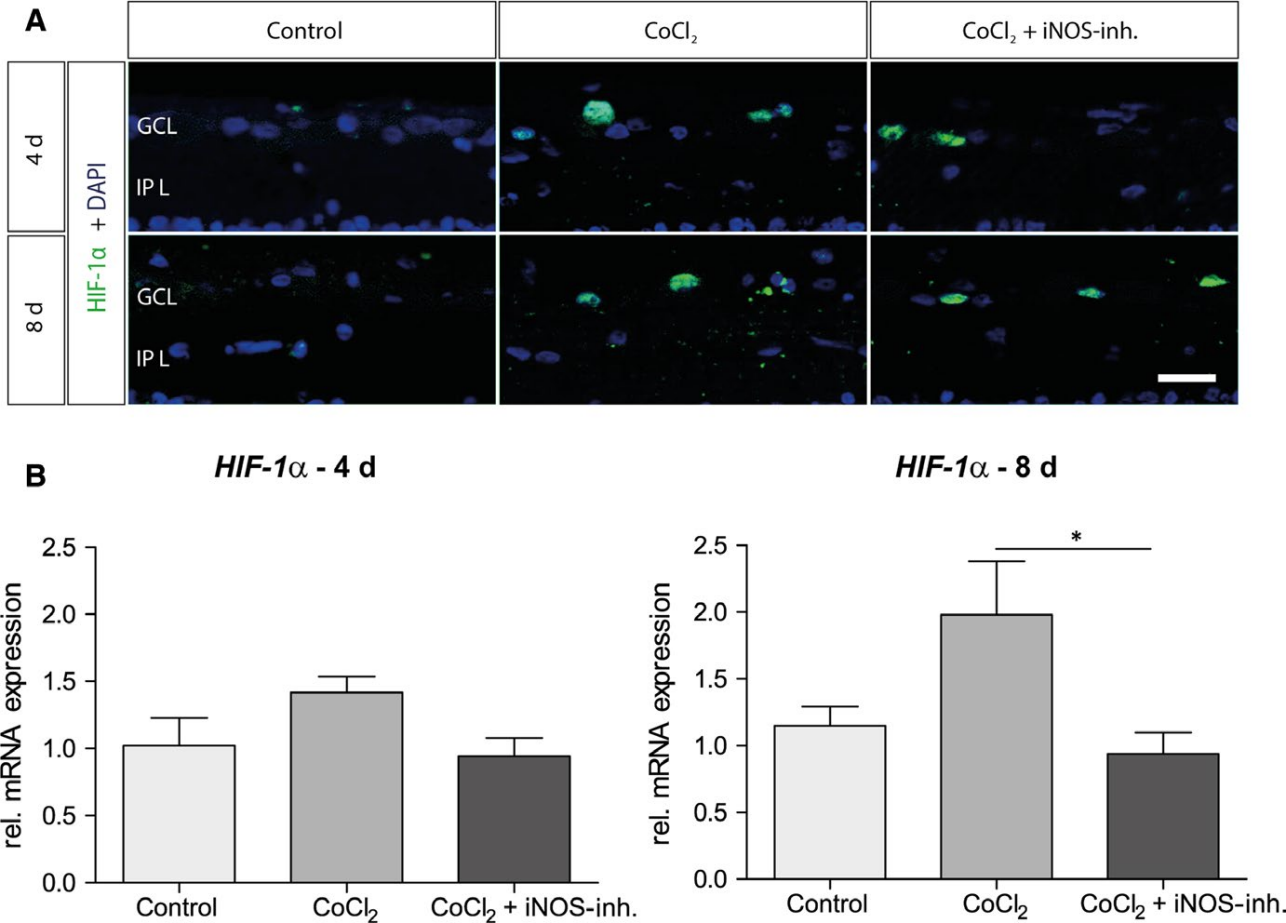
iNOS‐inhibitor mediated effect on hypoxia‐induced HIF‐1α expression. A, Representative pictures of HIF‐1α staining in the ganglion cell layer. HIF‐1α^+^ cells are marked in green and cell nuclei in blue. The addition of CoCl_2_ induced an increase or stabilization of the alpha subunit of the transcription factor. Under treatment with the iNOS inhibitor, the HIF‐1a levels were not lower compared with the CoCl_2_ group after 4 d. In addition, at 8 d of cultivation, comparable amounts of HIF‐1α were detected in CoCl_2_ and in the 1400W‐treated group. B, After 4 d, no significant differences in *HIF‐1α* mRNA expression were observed. At 8 d, the relative mRNA expression by CoCl_2_ was twofold increased and could be lowered to the initial level by treatment with 1400W. Abbreviations: GCL, ganglion cell layer; IPL, inner plexiform layer. Scale bar = 20 µm. All data are shown as mean ± SEM; *P < .05

Histologically, an HIF‐1α signal could be observed after 4 days in the CoCl_2_ group, which seemed to be only slightly reduced after 4 days under treatment with iNOS‐inhibitor 1400W (Figure 2A). No HIF‐1α^+^ cells were noted in the control group at 4 and 8 days. CoCl_2_ retinae still showed HIF‐1α signals later on as well as the CoCl_2_ + iNOS‐inh. ones, although just a few.

With the 4‐day‐groups no significant changes of the *HIF‐1α* mRNA level were observable (Figure 2B). After 8 days of cultivation, mRNA expression of the *HIF‐1α* was increased 1.9‐fold (*P* = .061) in the CoCl_2_ group. 1400W significantly reduced mRNA expression (*P* = .037; Figure 2B).

### 3.2 Influence of 1400W on iNOS and HSP70 expression

HIF‐1 is known to induce transcription of more than 60 genes, including *vascular enothelial groth factor (VEGF*; data not shown) and *iNOS.* In order to check whether this is the case in our model, we also analysed the mRNA expression of those markers.

**Figure 3:**
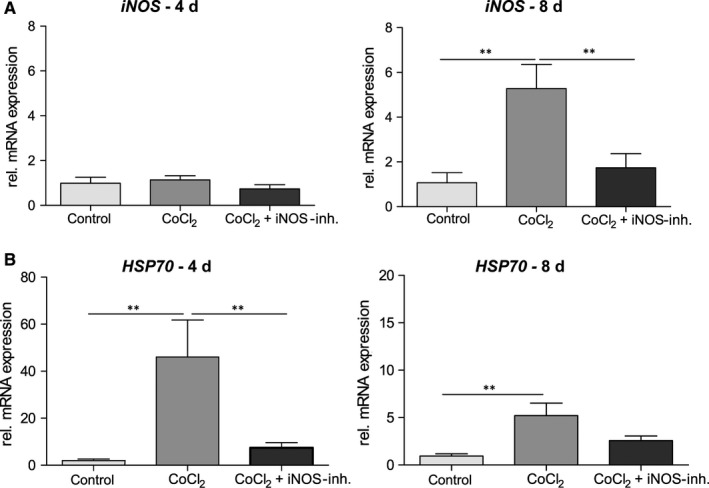
mRNA expression of the HIF‐1α target genes. A, CoCl_2_ had a significant effect on *iNOS* mRNA expression after 4 d and the additional treatment with 1400W only lead to a small decrease in mRNA. After 8 d, CoCl_2_ induced a fourfold increase in *iNOS* mRNA expression, this could be prevented by the 1400W treatment. B, After 4 d, a massive increase in mRNA expression of *HSP70* was observed in the CoCl_2_ retinas, which was significantly reduced by treatment with 1400W. After 8 d, a significantly increased expression was still observed, but not as pronounced as at the previous time. However, the protective effect of the inhibitor was still detectable after 8 d. All data are shown as mean ± SEM; **P < .01

Investigating of the mRNA expression of the *iNOS* revealed no alterations in the CoCl_2_ group and a non‐significant twofold reduction of the mRNA in 1400W‐treated group after 4 days. In the later time point, a significant (fourfold, *P* = .0036) mRNA increase by CoCl_2_ was observed, which was prevented by 1400W (*P* = .0085; Figure 3B). This effect confirmed the influence of 1400W in this retinal degeneration model.

Another marker of cellular stress is the heat shock protein 70 (HSP70). The inducible isoforms of HSP70 perform important functions, such as ensuring correct protein folding under stress conditions and can therefore also serve as markers for such stress. In the CoCl_2_ group, a strong (38.6‐fold, *P* = .001) increase of the relative mRNA expression of *HSP70* was observed at the first observation time (Figure 3B). 1400W treatment reduced the relative expression to a 7.4‐fold increase (*P* = .004). After 8 days, a less prominent, but still significant, 5.2‐fold induction (*P* = .0077) of mRNA was detected, which was also decreased by the iNOS‐inhibitor (2.5‐fold).

### 3.3 iNOS‐inhibitor mediates RGC protection via HIF‐1α regulation

**Figure 4:**
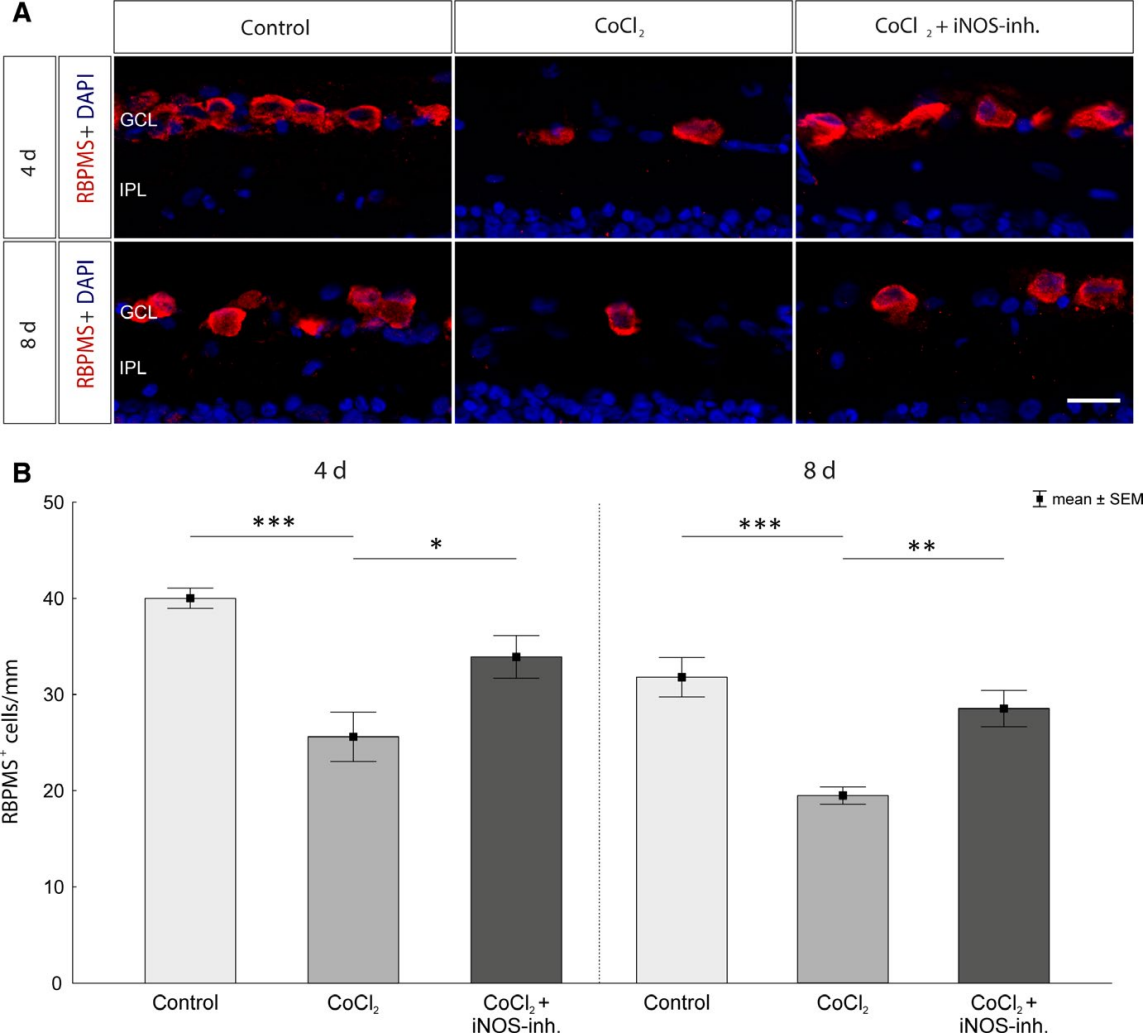
Rescue of retinal ganglion cells after CoCl_2_‐induced degeneration. A, Representative images of the immunohistological staining. RGCs were stained with an antibody against RBPMS (red) and cell nuclei with DAPI (blue). A significant loss of RGCs in the untreated degeneration groups (CoCl_2_) was observed over the cultivation period of 4 and 8 d. B, After 4 d, neuroprotection of the RGCs was observed by treatment with the iNOS‐inhibitor compared with the CoCl_2_ group. Even after 8 d of cultivation, a protection of the RGCs by 1400W could be noticed. The retinae of the treatment groups contained significantly more RGCs than the untreated retinae. GCL, ganglion cell layer; IPL, inner plexiform layer. Scale bar = 20 µm. All data are shown as mean ± SEM; *P < .05; **P < .01; ***P < .001

As first retinal cells, the CoCl_2_‐induced degeneration of RGCs was investigated. For this purpose, they were immunohistochemically stained with the specific antibody against RBPMS (Figure 4A). After 4 days, a significant loss of RGCs was observed compared to controls (control: 40.2 ± 1.0 cells/mm; CoCl_2_: 25.6 ± 2.5 cells/mm, *P* = .0002; Figure 4B). This loss could be counteracted by treatment with 1400W. In the retinae of the treatment group significantly more RGCs were detected compared with the untreated group (CoCl_2_ + iNOS‐inh.: 33.9 ± 2.2 cells/mm, *P* = .021) and also no significant difference to the control group was noticed (*P* = .109). After 8 days of cultivation a significant decline of the RGCs in the CoCl_2_‐treated retinae was also observed (control: 31.7 ± 2.0 cells/mm; CoCl_2_: 19.4 ± 0.9 cells/mm, *P* = .0002). Treatment with the iNOS‐inhibitor significantly reduced this effect, as these retinae contained significantly more RGCs than the untreated ones (CoCl_2_ + iNOS‐inh.: 28.5 ± 1.8 cells/mm, *P* = .002). Furthermore, there was no significant difference in the number of RGCs between the treatment and the control group (*P* = .374; Figure 4B).

### 3.4 CoCl_2_ induced irreversible degeneration of microglia and decreases their activity

**Figure 5:**
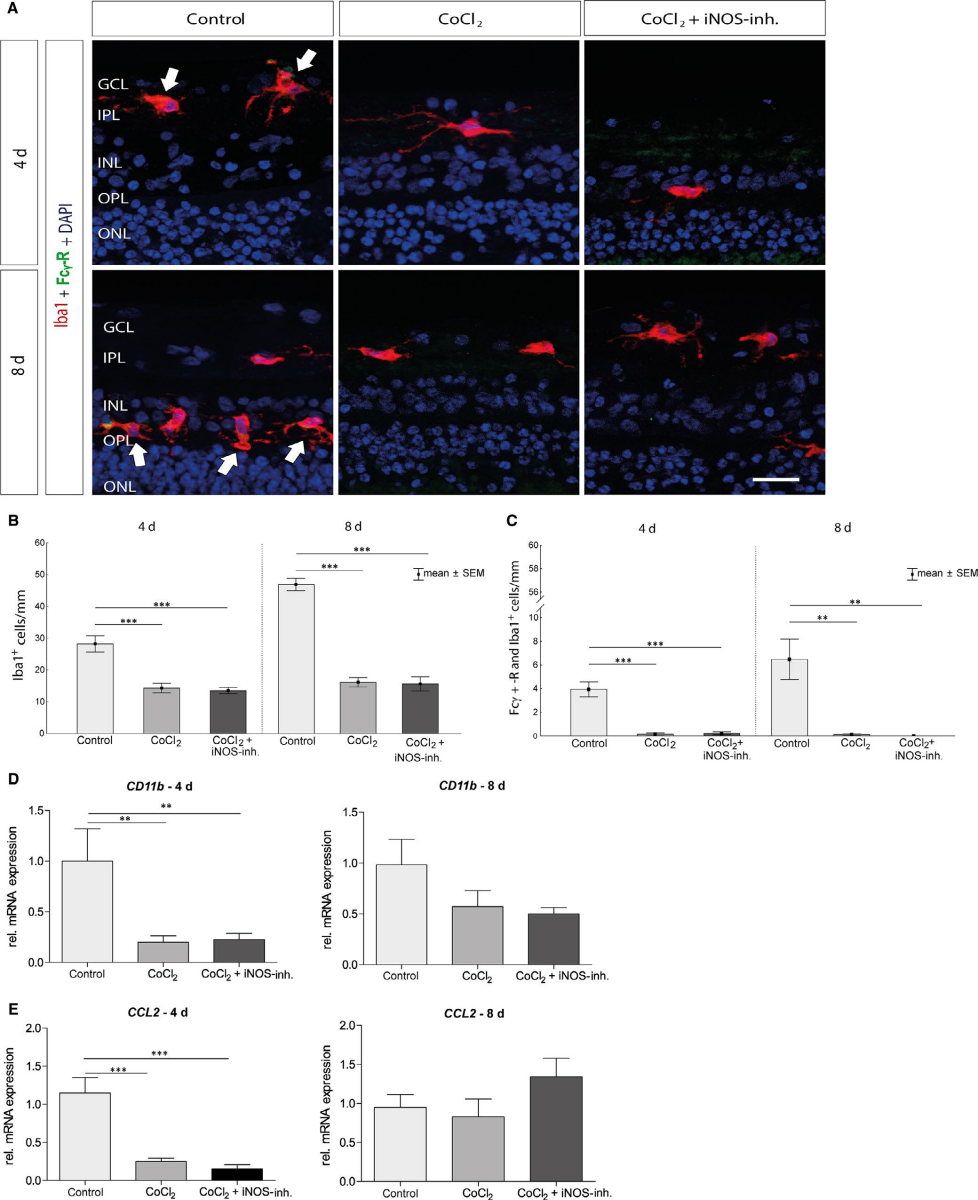
CoCl_2_ degeneration irreversibly reduces the microglia. A, Microglia were stained with anti‐Iba1 (red) on day 4 and 8 of cultivation. Fcy‐R (green, arrows) in combination with Iba1 served as an activity marker of the microglia. Cell nuclei are shown in blue. B, The addition of CoCl_2_ triggered a significant loss of microglia in comparison with controls at both times. In the 1400W‐treated group also, significantly fewer microglia were present than in the control. C, In addition, the number of activated microglia was significantly lower in the CoCl_2_ group than in the control group after 4 and 8 d. Again, treatment with the iNOS‐inhibitor had no protective effect. D, The relative *CD11b* mRNA expression was also significantly reduced in the CoCl_2_ group, after 4 and 8 d of cultivation. The 1400W treatment did not result in an improvement compared with control at both times. E, The analysis of the relative *CCL2* mRNA expression showed that it was significantly reduced after 4 d in retinae of the CoCl_2_ group. Again, mRNA expression could not be altered by the 1400W. After 8 d of cultivation, no differences between the groups were observed. GCL, ganglion cell layer; INL, inner nuclear layer; IPL, inner plexiform layer; ONL, outer nuclear layer; OPL, outer plexiform layer. Scale bar = 20 µm. All data are shown as mean ± SEM; **P < .01; ***P < .001

Immunohistochemical staining was used to examine the total population of microglia in the retina (anti‐Iba1 (red); Figure 5A). Activated microglia exhibited a strongly increased expression of the Fcy‐receptor; thus, all Iba1^+^ and Fcy‐R^+^ cells (green) were evaluated as active microglia. As previously observed,^3^ after 4 days incubation with CoCl_2_ leads to a significant loss of the microglia population compared with controls (control: 28.2 ± 2.5 cells/mm; CoCl_2_: 14.3 ± 1.4 cells/mm; *P* = .0002). This could not be prevented by the 1400W treatment (13.5 ± 1.0 cells/mm; *P* = .0001). After 8 days, more Iba1^+^ cells were found in the control group (46.90 ± 1.93 cells/mm), but also here an irreversible loss of microglia was observed under CoCl_2_ treatment (CoCl_2_: 16.1 ± 1.4 cells/mm; *P* = .0002; CoCl_2_ + iNOS‐inh.: 15.6 ± 2.1 cells/mm; *P* = .0002; Figure 5B).

The analysis of the activated microglia showed similar results. The CoCl_2_ retinae had significantly less activated microglia on both observation days than the control (4 days: control: 3.9 ± 0.6 cells/mm; CoCl_2_: 0.14 ± 0.1 cells/mm, *P* = .0001; CoCl_2_ + iNOS‐inh.: 0.22 ± 0.1 cells/mm, *P* = .0001 and 8 days: control: 6.4 ± 1.7 cells/mm; CoCl_2_: 0.12 ± 0.1 cells/mm, *P* = .001; CoCl_2_ + iNOS‐inh.: 0.0 ± 0.0 cells/mm, *P* = .001; Figure 5C).

In addition, the relative mRNA expression of *CD11b*, another marker of the microglia, and *CCL2,* a CC chemokine, which regulates the activation and recruitment of macrophages, was tested. Here, a down‐regulation of immunocompetent cells was also clearly evident. After 4 days, the relative mRNA expression of *CD11b* (*P* = .0094; Figure 5D) and *CCL2* (*P* = .0001; Figure 5E) was significantly reduced in the CoCl_2_ group. The expression could not be increased by 1400W treatment (*CD11b*: *P* = .0077 and *CCL2:* *P* = .0001; Figure 5D + E). After 8 days of cultivation, there was still a reduction of *CD11b* mRNA, which was not significant (*P* = .0512) (Figure 5D). And there were no differences in *CCL2* mRNA expression (Figure 5E).

### 3.5 No rescue of amacrine cells through iNOS‐inhibitor treatment

**Figure 6:**
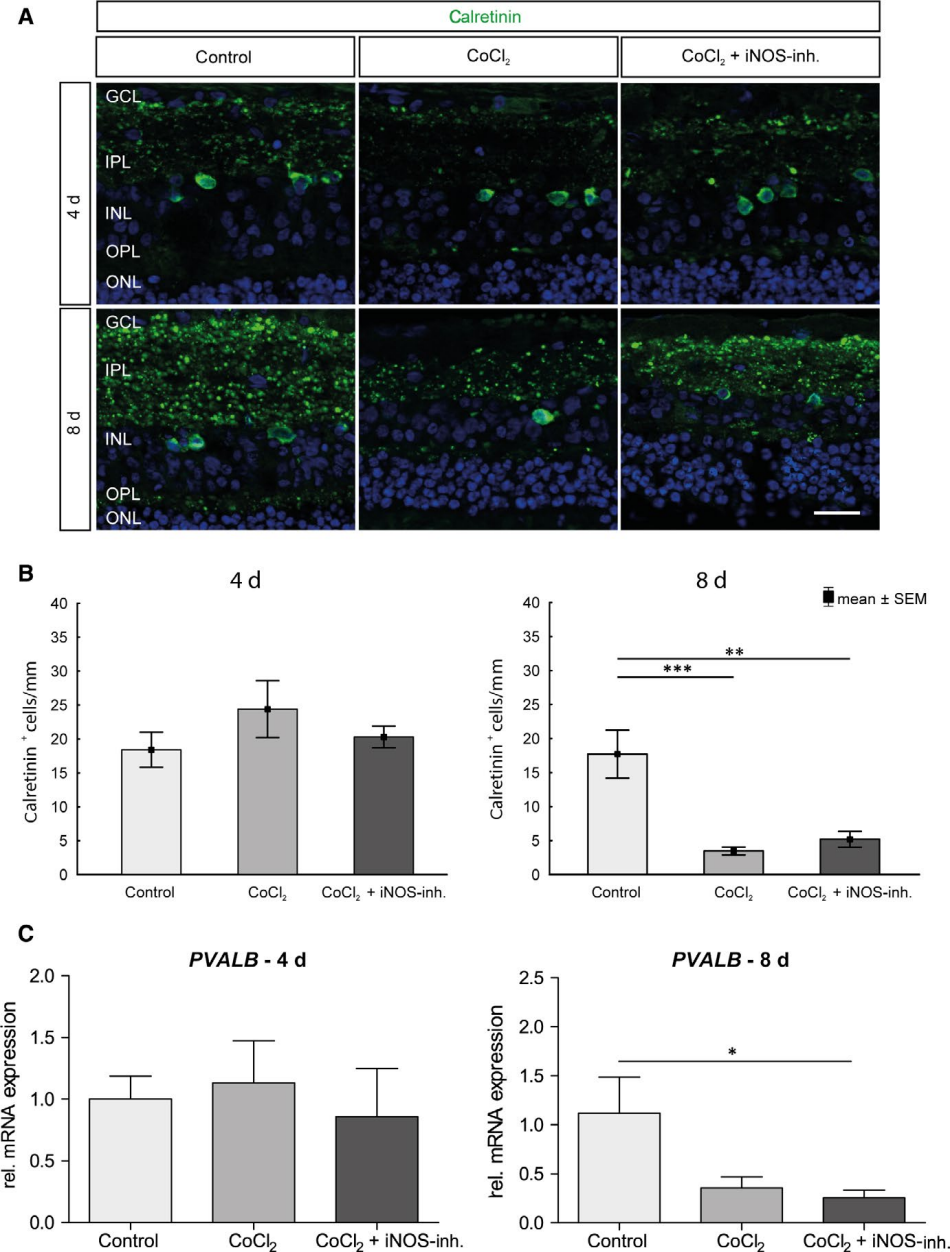
No positive influence of 1400W treatment on amacrine cells. A, Representative pictures of amacrine cell population in retinal explants. Amacrine cells were labelled with antibodies against calretinin (green). Nuclei were stained with DAPI (blue). B, On day 4, no significant differences could be observed between the groups. On day 8, a significant loss of amacrine cells in retinae of the CoCl_2_ group was detected. The iNOS inhibitor treatment could not protect the amacrine cells. C, No differences in the relative expression of *PVALB* mRNA were observed on day 4. On day 8, the relative expression of *PVALB* was reduced in all groups compared with the control and significantly in comparison with the iNOS‐inh. treatment. GCL, ganglion cell layer; INL, inner nuclear layer; IPL, inner plexiform layer; ONL, outer nuclear layer; OPL, outer plexiform layer. Scale bar = 20 µm. All data are shown as mean ± SEM. *P < .05; **P < .01; ***P < .001

The degenerative influence of CoCl_2_ and the potential protective effect of 1400W on amacrine cells were investigated using relative *Parvalbumin* (*PVALB*) mRNA expression and immunohistochemical analyses of the calretinin‐positive cell population. PVALB and calretinin are assigned to the group of calcium‐binding proteins and can be detected in amacrine cells.^29^ On day 4, no significant difference in the number of calretinin‐positive amacrine cells could be observed between the individual groups (Figure 6A+B). At day 8, the number of calretinin^+^ cells was decreased and this loss was still present after treatment with the iNOS‐inhibitor (control: 17.7 ± 11.1 calretinin^+^ cells/mm; CoCl_2_: 3.4 ± 1.8 calretinin^+^ cells/mm, *P* = .0003; CoCl_2_ + iNOS‐inh.: 5.1 ± 3.6 calretinin^+^ cells/mm, *P* = .001). Also, no significant difference in relative *PVALB* expression could be measured between the groups after 4 days (Figure 6C). At day 8, a 2.8‐fold decrease in relative *PVALB* expression was noted in the CoCl_2_ group compared to the controls (*P* = .0518), which could not be eliminated by treatment with iNOS‐inhibitor (4‐fold decrease, *P* = .0265; Figure 6C).

### 3.6 Protection of bipolar cells by inhibition of iNOS

**Figure 7:**
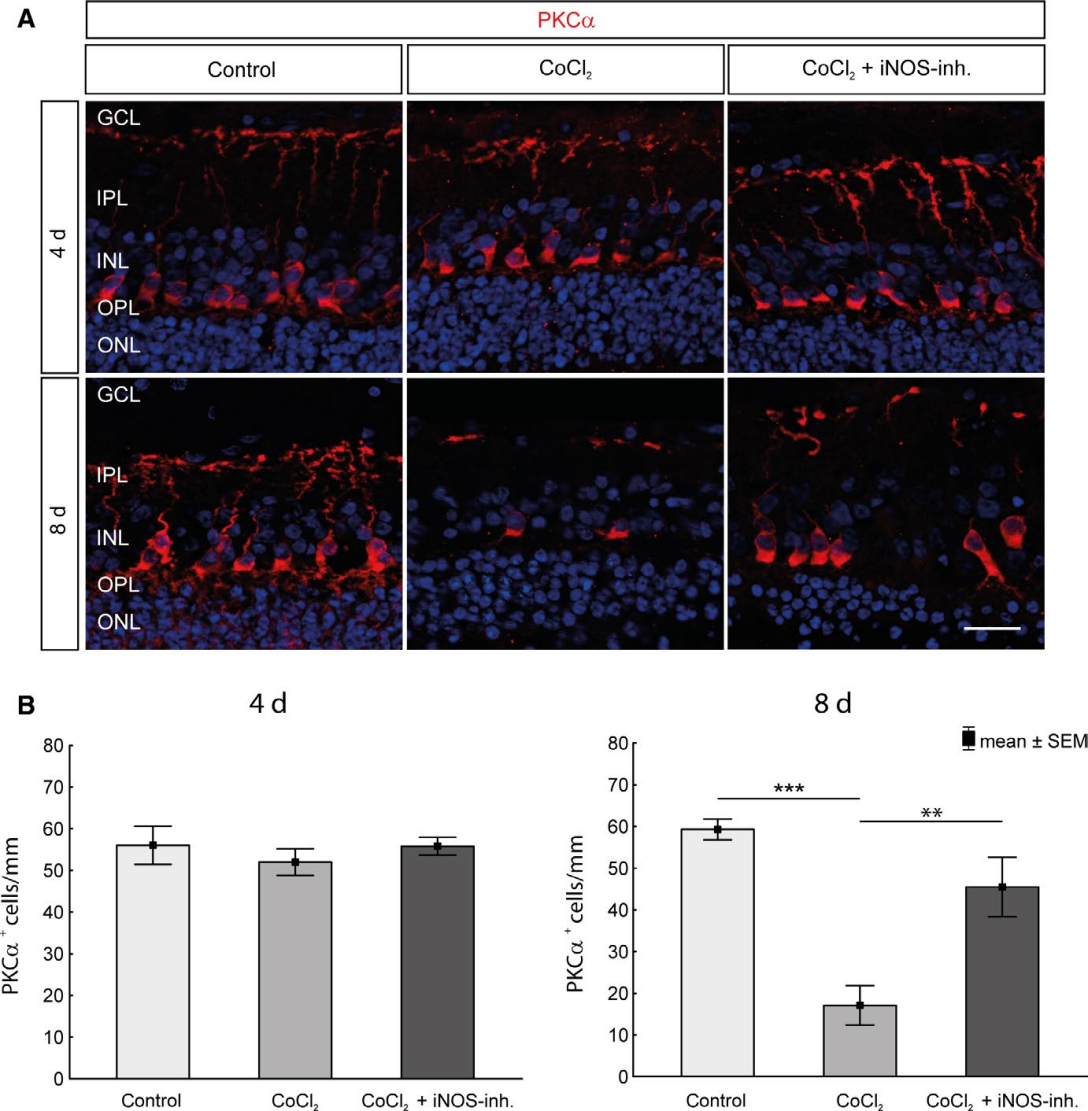
Protection of bipolar cells against CoCl_2_‐induced degeneration. A, Nuclei were visualized with DAPI staining (blue), and bipolar cells were stained with an antibody against PKCα (red). B, No significant loss of bipolar cells in the untreated degeneration groups could be detected during the cultivation period of 4 d. After 8 d, a significant loss was observed. However, protection of the bipolar cells by 1400W was observed. The retinae of the treatment group contained significantly more bipolar cells than the untreated retinae. GCL, ganglion cell layer; INL, inner nuclear layer; IPL, inner plexiform layer; ONL, outer nuclear layer; OPL, outer plexiform layer. Scale bar = 20 µm. All data are shown as mean ± SEM; **P < .01; ***P < .001

Examination of the bipolar cells of the retina was another important point in this project. Bipolar cells collect the information of the photoreceptors, process and forward those to the RGCs. Using the marker PKCα, the quantity of bipolar cells in the retinal explants was investigated histologically (Figure 7A). No differences were found after 4 days. However, the degenerative effect of CoCl_2_ after 8 days was characterized by a significant decrease (control: 59.2 ± 7.8 PKCα^+^ cells/mm; CoCl_2_ 17.1 ± 14.9 PKCα^+^ cells/mm; *P* = .000134) of the bipolar cells, which was prevented by the 1400W treatment (45.5 ± 7.1 PKCα^+^ cells/mm; *P* = .0017; Figure 7B).

## 4 DISCUSSION

The degeneration processes of many retinal diseases have not been fully investigated yet. In order to understand the pathological changes, reliable models are needed, in which eye diseases can be simulated. In this study, we present a promising option in which not only the degeneration process in retinal tissue could be simulated in a straight‐forward and standardized way, but also drug therapy testing could be performed. The advantages of these ex vivo cultures are obvious: The anatomy of pig eyes compared to human eyes is morphologically and physiologically more similar to that of rodents.^6^ In addition, the complex structure of the retina is preserved and the reproducibility is much higher as a higher number of samples can be obtained.^2^, ^30^

Retinopathy is the main cause of blindness and visual impairment in people of all ages. The pathogenesis of retinopathy is caused by numerous factors. Considering only the ‘hypoxic factors’, these include changes that contribute to oxidative stress, like increased nitric oxide and superoxide production, changes in the expression of various isoforms of nitric oxide synthase, or the endogenous antioxidant system.^31^ Hypoxia is a main trigger of the pathogenic mechanism in retinal diseases. This is a multifactorial, dynamic process involving oxidative stress, inflammation, and cell death as well as the activation of regenerative mechanisms dependent on the hypoxia inducible transcription factor HIF‐1α.^32^, ^33^ HIF‐1α is one of the key regulatory components in the cell's hypoxia reaction system and in turn increases the transcription of numerous other genes.^34^

Neurodegeneration consists of a cascade of different processes; one of the pathways is the regulation of NO levels. NO is synthesized from l‐arginine by a class of haeme‐dependent enzymes called nitric oxide synthases and is an important signalling molecule that mediates a variety of physiological processes, including neurotransmission, vasodilation, and host cell defence.^20^, ^21^ In the retina, NO has a special function as a regulator of visual adaptation. It controls the light reactions in all retinal neuron classes. Further in rods, cones, bipolar, and ganglion cells certain ionic conductivities are activated by the neurotransmitter. The light‐dependent gap‐transition coupling in the inner and outer plexiform layer is also influenced by NO.^35^

On the other hand, iNOS is an inducible enzyme which only acts immunologically in pathological conditions by endotoxins, inflammation, certain cytokines, and hypoxia. Once induced, iNOS produces large amounts of NO for long periods of time. Therefore, NO is converted into NO_2_, nitrite, and free radicals to induce pathophysiological actions, such as optic nerve degeneration and posterior retinal degeneration lesion, which lead to glaucoma, retinopathy, AMD, cataracts, and uveitis.^19^

iNOS‐inhibitors were tested as therapeutic agents for neurodegenerative diseases and pain since years. Most of the iNOS‐inhibitors described so far were analogues of the substrate l‐arginine. In recent developments, isothioureas and bisisothioureas have been described as potent and selective inhibitors of human iNOS isocyanins.^36^ 1400W (*N*‐(3‐(Aminomethyl)benzyl)acetamidine is in turn a further development, which is synthesized based on bisisothiourea.^37^, ^38^

The aim of this project was to demonstrate possible therapeutic effects by inhibiting the inducible nitric oxide synthase in a CoCl_2_‐induced retinal explants model.

By treating cells or organotypic explant with CoCl_2_, hypoxia can be induced ex vivo and the pathological mechanism simulated.^3^, ^34^, ^39^ CoCl_2_, similar to hypoxia, prevents the degradation of the α‐subunit of hypoxia‐inducible factor and thus mediates its stabilization.^40^ With our CoCl_2_ induced retinal degeneration model, we already proved the neuroprotective effect of hypothermia.^4^ Here, we specifically examined the influence of the inhibition of iNOS on the course of degeneration.

CoCl_2_ induced hypoxia led to a significant loss of RGCs after 4 and 8 days, which was counteracted by treatment with 1400W. As described before, incubation with CoCl_2_ induces apoptosis, especially in RGCs.^3^, ^41^ We have already shown that CoCl_2_ not only mimics hypoxia by stabilizing HIF‐1α, but also leads to an elevated ROS level by disrupting the mitochondrial respiratory chain.^4^, ^33^ The mechanism behind the 1400W mediated protection of RGCs is possibly based on reduced NO production and thus on the prevention of apoptosis. In our model, it could be observed that treatment with the iNOS inhibitor significantly reduced the amount of *iNOS* and *HIF‐1α* mRNA expression.^42^ It is known that hypoxia induces HIF‐1α and its target genes, such as VEGF and iNOS, in many tissues.^43^ The pathophysiological accumulation of these factors has been associated with neuronal death under hypoxic‐ischaemic conditions. Moreover, overproduction leads to increased extracellular accumulation of glutamate and inflammatory cytokines, which damage the neurons.^44^ Another marker for cellular stress is HSP70, whose expression can be induced by HIF‐1α.^45^ In CoCl_2_‐stressed retinas, the mRNA expression of *HSP70* was strongly elevated.^46^ HSPs are chaperones that are up‐regulated during cellular stress. Their task is to prevent misfolded proteins and protein aggregation. Thus, HSPs plays an important role for the accumulation and function of HIF‐1α.^47^ These signalling pathways were blocked by treatment with the iNOS inhibitor. A reduction of both *HIF‐1α* and *HSP70* mRNA expression was observed, both after 4 and 8 days.

Sennlaub et al. demonstrated that Müller cells express iNOS in vitro, and they may therefore be a major source of iNOS expression. Furthermore, other cell types, such as amacrine, horizontal, bipolar and microglial cells, contribute to the NO production during ischaemic proliferative retinopathy.^48^ Microglia are an essential mediator of neuroinflammation in many neurological disorders and are susceptible to HIF‐1α.^49^ Likewise, there are reports that describe that besides oligodendrocytes, the microglia are the glial cell types most susceptible to hypoxia^50^ and are extremely sensitive to their microenvironment.^44^, ^51^ We observed the same effects in our ex vivo model (Figure 5). Incubation with CoCl_2_ has massive degenerative effects on microglia.^3^ The inhibition of iNOS is not an obvious way to increase microglia number in this case. Based on the type of cultivation, with just a piece of the retina, no interaction with the optic nerve and the retinal pigment epithelium, the microglia are more turned to pro‐inflammatory M1 subtypes. iNOS is a marker which is only produced by M1 microglia/macrophages.^52^ The reduction of iNOS inhibited the microglia. Furthermore, high levels of VEGF could reduce the number of M1 microglia as well, which is already shown in an ischaemic brain rat model.^53^ Therefore, the treatment with the iNOS inhibitor had no beneficial effect on the microglia number in contrast to the already published treatment with hypothermia.^4^ Other publications describe a balance between harmful and protective factors in the retina after hypoxia. It is therefore conceivable that microglia react early to hypoxic stress but are down‐regulated after 4 or 8 days to protect the retina.^44^, ^54^, ^55^

Inner layers of the retina are known to be most sensitive to hypoxic challenges, whereas the outer retina is more resistant to hypoxic stress.^56^, ^57^ Investigations of other cell types of the inner retina revealed that CoCl_2_ led to a loss of calretinin positive amacrine cells and PKCα‐positive bipolar cells after 8 days, which was also described before.^3^ While the bipolar cells could be protected by 1400W treatment, the therapy had no protective effect on amacrine cells. Bipolar cells are the only neurons that connect the outer retina to the inner retina. Since 1400W had a neuroprotective effect on bipolar and RGCs, but not on amacrine cells, it can be anticipated that the degeneration process triggered by CoCl_2_ is different in these diverse neuronal cell types. Amacrine cells are interneurons in the retina and synaptically active in the inner plexiform layer. They are inhibitory neurons that interact with RGCs and bipolar cells.^58^ Amacrine cells express receptors for glutamate released from bipolar cells. Glutamate, a neurotransmitter, is important for the physiology of amacrine cells. However, over‐activation of glutamate receptors under pathological conditions, like hypoxia or retinal ischaemia, causes amacrine cell death.^59^, ^60^ Furthermore, increased production of NO is believed to mediate neuronal injury caused by glutamate acting on NMDA receptors.^44^, ^61^ This might be one mechanism how CoCl_2_ induced loss of amacrine cells and why it could not be prevented by the iNOS‐inhibitor.

## 5 CONCLUSION

The iNOS‐inhibitor 1400W led to neuroprotective effects in the retina and many but not all cell types responded with an increased survival rate to the therapy. This allowed us to prove the neuroprotective properties of 1400W and at the same time prove that ex vivo organ cultures are very suitable for drug therapy testing.

## CONFLICT OF INTEREST

The authors confirm that there are no conflicts of interest.

## AUTHORS' CONTRIBUTIONS

AMM‐B, FH and LH cultivated retinal explants and performed the histological examinations of the explants. SK supported the statistical analysis of the data. JH performed the qRT‐PCR examination and was a major contributor in writing the manuscript. SS and SCJ revised the manuscript, planed and designed the study. All authors read and approved the final manuscript.

## Data Availability

All data generated or analysed during this study are included in this published article.
